# 1,25(OH)_2_D_3_ Inhibited Ferroptosis in Zebrafish Liver Cells (ZFL) by Regulating Keap1-Nrf2-GPx4 and NF-κB-hepcidin Axis

**DOI:** 10.3390/ijms222111334

**Published:** 2021-10-20

**Authors:** Ke Cheng, Yanqing Huang, Chunfang Wang

**Affiliations:** 1 Hubei Provincial Engineering Laboratory for Pond Aquaculture, The College of Fisheries, Huazhong Agricultural University, Wuhan 430070, China; chengke714@outlook.com; 2 Engineering Research Center of Green Development for Conventional Aquatic Biological Industry in the Yangtze River Economic Belt, Ministry of Education, Wuhan 430070, China; 3 East China Sea Fisheries Research Institute, Chinese Academy of Fishery Sciences, Shanghai 200090, China; hyqrich@163.com

**Keywords:** ferroptosis, 1,25(OH)_2_D_3_, zebrafish liver cells, Keap1–Nrf2–GPx4, NF-κB–hepcidin

## Abstract

Ferroptosis is a kind of iron-dependent programed cell death. Vitamin D has been shown to be an antioxidant and a regulator of iron metabolism, but the relationship between vitamin D and ferroptosis is poorly studied in fish. This study used zebrafish liver cells (ZFL) to establish a ferroptosis model to explore the effect of 1,25(OH)_2_D_3_ on cell ferroptosis and its mechanism of action. The results showed that different incubation patterns of 1,25(OH)_2_D_3_ improved the survival rate of ZFL, mitigated mitochondrial damage, enhanced total glutathione peroxidase (GPx) activity, and reduced intracellular reactive oxygen species (ROS), lipid peroxidation (LPO), and malondialdehyde (MDA), as well as iron ion levels, with the best effect at 200 pM 1,25(OH)_2_D_3_ preincubation for 72 h. Preincubation of ZFL at 200 pM 1,25(OH)_2_D_3_ for 72 h downgraded *keap1* and *ptgs2* gene expression, increased *nrf2, ho-1, fth1, gpx4a,b* expression, and lowered the expression of the *nf-κb p65,il-6,il-1β* gene, thus reducing the expression of *hamp1.* The above results indicate that different incubation patterns of 1,25(OH)_2_D_3_ have protective effects on ferroptosis of ZFL induced by ferroptosis activator RSL3 and 1,25(OH)_2_D_3_ can inhibit ferroptosis of ZFL by regulating Keap1–Nrf2–GPx4 and NF-κB–hepcidin axis.

## 1. Introduction

In recent years, in addition to the typical cell apoptosis, autophagy, pyroptosis, and necrosis, researchers also found a new form of regulatory cell death—ferroptosis. Ferroptosis was first discovered by Dixon et al. [1] in the study of the death process of cancer cells. It refers to a form of regulatory cell death due to lethal lipid peroxidation. This new cell death mode is dependent on iron ions and is therefore named ferroptosis. Cell ferroptosis undergoes unique morphological changes, such as disruption of cellular junctions, mitochondrial shrinkage, cristae shrinkage, and rupture of mitochondrial outer membrane, but does not exhibit chromatin condensation, and cell membrane rupture [2]. In terms of physiology and biochemistry, ferroptosis will lead to the increase in intracellular iron ion level, the generation of a large amount of ROS, the depletion of glutathione peroxidase 4 (GPx4), and the accumulation of lipid metabolites [3,4].

One of the key regulatory mechanisms of ferroptosis is the Nrf2–GPx4 regulatory pathway. Nuclear factor erythroid 2-related factor 2, Nrf2, in addition to its traditional antioxidation and anti-inflammatory effects, can also regulate intracellular Fe^2+^ and inhibit ferroptosis [5]. Normally, Nrf2 is inactive. Once ROS in the cells accumulates excessively, Nrf2 is activated, thereby activating downstream antioxidant enzymes as an antioxidant and inhibiting cell ferroptosis [6]. Previous study has found that upregulation of Nrf2 promotes the transcription of downstream antioxidant proteins, such as GPx4, while downregulation of Nrf2 enhances the ferroptosis of liver cells induced by ferroptosis activator [2]. GPx4 can convert toxic lipid peroxides into nontoxic lipid alcohols and decompose H_2_O_2_ into H_2_O, which has the effect of oxidative damage repair [7]. It was found that the GPx4 knockout mouse survived for only 7.5 days [8]. The content of superoxide anions together with hydroxylated free radicals in mice treated with GPx4 inhibitors increased rapidly, and ferroptosis was observed in cells [9]. It has also been found in cancer research that the silencing of the *gpx4* gene promoted ferroptosis of lung cancer cells [10]. In addition, Xu et al. [11] confirmed that GPx4 alleviated the negative effects of the lipid peroxidation response and ferroptosis on activated T regulatory cells. Therefore, the antioxidant enzyme GPx4 is a crucial regulatory protein for ferroptosis and Nrf2–GPx4 is conceived to be a key signaling pathway for regulating ferroptosis.

In addition, iron concentration in cells can also affect the occurrence of ferroptosis, and hepcidin is a critical factor in maintaining iron ion balance in animal cells. The regulation of iron homeostasis by hepcidin was first observed in mice by Pigeon et al. [12]. Subsequently, Hentze et al. [13] proposed the mechanism by which hepcidin regulates iron metabolism in mammals: hepatocytes are responsible for sensing ferroportin saturation, as well as iron storage status, and release hepcidin accordingly. When iron is overloaded, the expression of hepcidin gene (*hamp*) is highly upregulated, which causes the ferroportin on the membrane to degrade and, thus, inhibit the output of iron. When iron is deficient, hepcidin gene expression is suppressed, and iron stored in liver cells is transported by ferroportin and released into the circulation. Previous study has shown that interleukin 6 (IL-6) and interleukin-1β (IL-1β) are precursors of hepcidin, and both are regulated by the nuclear factor kappa B (NF-κB) signaling pathway, which promotes inflammatory responses and participates in immune responses [14]. In fish, Shike et al. [15] first isolated and identified hepcidin in a cross of Morone chrysops × Morone saxatilis. Later, Rordigues et al. [16] exposed sea bass to iron overload and found that the hepcidin gene in the liver was upregulated, but no change was detected in the iron deficiency environment. The barbel steed (*Hemibarbus labeo*) quickly responded to iron overload in the environment by upregulating the expression of hepcidin gene [17]. Iron overload induced the expression of hepcidin gene in turbot [18]. These results indicate that hepcidin can regulate iron metabolism in fish. However, to our knowledge, there is no systematic and complete study on the mechanism of hepcidin regulating iron content in fish.

Vitamin D (VD) is a nutrient necessary for the growth of plants and animals. In animals, VD must be converted into its active form to function. 1,25-Dihydroxyvitamin D_3_ (1,25(OH)_2_D_3_) is one of the active metabolites of vitamin D_3_ (VD_3_) and is also known as active vitamin D_3_ or calcitriol. 1,25(OH)_2_D_3_ is required to bind with vitamin D receptors (VDR) and retinoid X receptors (RXR) to form a ternary complex for biological effects [19]. In fish, VD_3_ was first recognized to regulate the metabolism and balance of calcium and phosphorus. However, recent studies have found that VD_3_ can also regulate innate immunity and exert antioxidant function [20,21,22,23,24,25,26,27]. Furthermore, VD has also been reported to regulate iron metabolism and to be a modulator of the hepcidin–ferroportin axis in humans and mammals [28]. Based on the previous experimental results from our laboratory, the supplementation of 1,25(OH)_2_D_3_ in cell culture medium reduced ROS production induced by lipopolysaccharide (LPS) and polyinosinic-polycytidylic acid (poly(I:C)) in yellow catfish head kidney macrophages, while supplementation of VD_3_ in diets significantly lowered the deposition of hemosiderin in yellow catfish spleen after *Edwardsiella ictaluri* challenge. Moreover, dietary VD_3_ affected the ‘iron ion transport’ signaling pathway in yellow catfish immune tissues based on the next-generation sequencing results [23,24,25]. However, information about the relationship between VD and ferroptosis has not been well documented. In the study of human acute kidney injury disease, Hu et al. [29] found that vitamin D receptor (VDR) deficiency significantly reduced the expression of GPx4 in both in vivo and in vitro experiments, and further luciferase reporter gene analysis showed that GPx4 was the target gene of transcription factor VDR. After the use of VDR activators, lipid peroxidation (a characteristic phenotype of ferroptosis) together with MDA levels were reduced, and these effects were thought to be related with the GPx4 signal pathway [29]. Bell [30] reported that, when human peripheral blood mononuclear cell lines (THP-1) were stimulated by higher concentrations of VD_3_ (10 and 100 nM), the expression of the hepcidin gene decreased. Although several studies in human and mammals have demonstrated the possible function of VD_3_ in iron metabolism, few studies have elucidated the association between VD and ferroptosis in fish.

Since liver is the iron storage organ of fish, in this study, the liver cell lines of model organism zebrafish and the ferroptosis activator RSL3 were used to establish the ferroptosis model of fish cells. The objective of this study was to investigate whether 1,25(OH)_2_D_3_ inhibits RSL3-induced ferroptosis in zebrafish liver cells by means of the Keap1–Nrf2–GPx4 and NF-κB–hepcidin axis.

## 2. Results

### 2.1. ZFL Mitochondrial Morphological Observation

**Figure 1:**
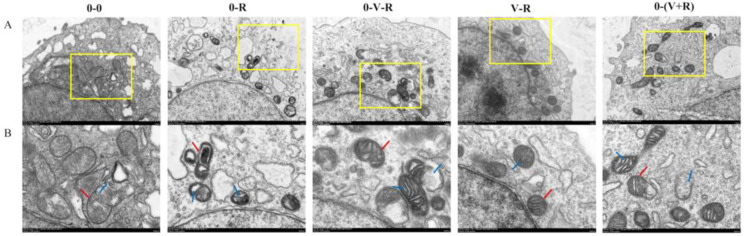
Effects of different incubation patterns of 1,25(OH)_2_D_3_ on morphological changes of ZFL mitochondria after being treated with 3 μM RSL3 for 6 h. Each group has two pictures, where picture (**B**) is an enlarged view of the yellow box area in picture (**A**), and the scale is marked on the bottom right of each picture. In (**B**), the red arrow indicates the mitochondrial membrane and the blue arrow indicates the cristae.

The effects of different incubation patterns of 1,25(OH)_2_D_3_ on the morphological changes of ZFL treated with 3 μM RSL3 for 6 h are shown in Figure 1. In the 0-0 group, the mitochondria of ZFL were normal ovals, with a clear bilayer membrane and normal cristae distribution. However, in the 0-R group, mitochondrial rupture occurred, the bilayer boundaries were blurred, and crista shrinkage resulted in a lot of empty bubbles in the mitochondria, implying that RSL3 could cause serious mitochondrial damage. Compared with the 0-R group, the ZFL in the 0-V-R, V-R, and 0-(V+R) groups had reduced mitochondrial damage. Especially in the V-R group, the mitochondrial morphology was similar to that of the 0-0 group. These results demonstrated that 1,25(OH)_2_D_3_ could reduce the mitochondrial damage caused by RSL3, and preincubation for 72 h has the best inhibitory effect.

### 2.2. Effects of 1,25(OH)_2_D_3_ on the Survival of ZFL

**Figure 2:**
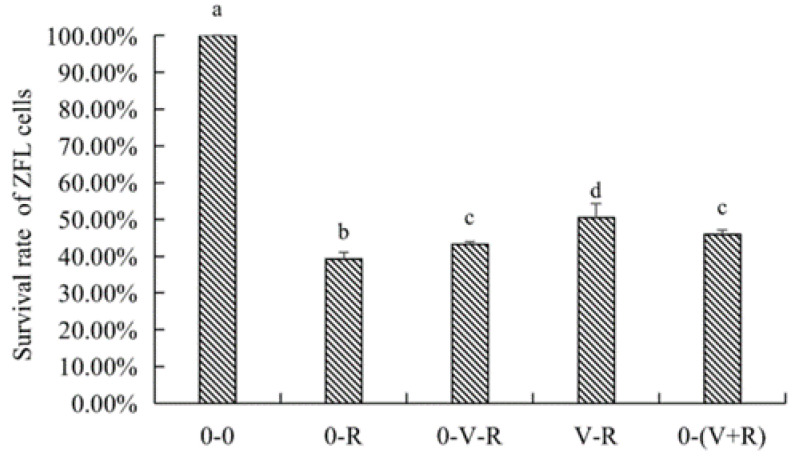
Effects of different incubation patterns of 1,25(OH)_2_D_3_ on survival rate of ZFL after being treated with 3 μM RSL3 for 6 h (*n* = 6). Error bars represent the standard error of each group. Means with different superscript letters (a, b, c, d) are significantly different (*p* < 0.05),while the same letter means no statistical difference between two groups.

The effects of different incubation patterns of 1,25(OH)_2_D_3_ on the survival rate of ZFL treated with 3 μM RSL3 for 6 h are shown in Figure 2. The survival rate of ZFL in the 0-R group was significantly lower than that in the 0-0 group (*p* < 0.05), indicating that 3 μM RSL3 was able to induce ZFL death. The ZFL survival rates of the 0-V-R, V-R, and 0-(V+R) groups were significantly improved (*p* < 0.05) compared to that in the 0-R group, wherein the ZFL survival rate of V-R group was significantly higher than that in the 0-V-R group (*p* < 0.05). No statistical difference was observed between the 0-V-R group and 0-(V+R) group (*p* > 0.05), connoting that, in the current study, 1,25(OH)_2_D_3_ preincubated for 72 h was more protective on RSL3-stimulated ZFL than that preincubated for 6 h. There was no difference in the effects of 1,25(OH)_2_D_3_ preincubated for 6 h and 1,25(OH)_2_D_3_ plus RSL3 co-incubated for 6 h on improved ZFL survival (*p* > 0.05).

### 2.3. Effects of 1,25(OH)_2_D_3_ on the Total GPx Activity, ROS, and MDA Level of ZFL

**Figure 3:**
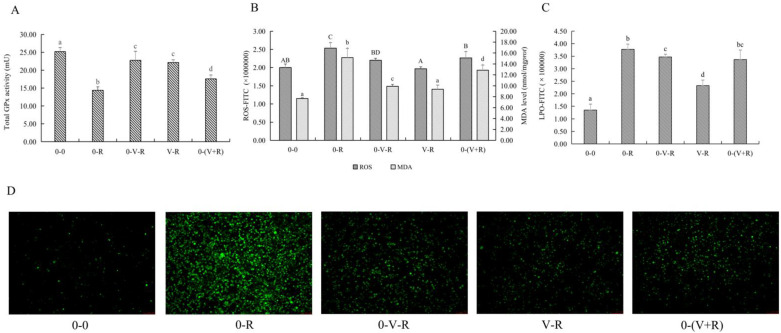
Effects of different incubation patterns of 1,25(OH)_2_D_3_ on total GPx activity (**A**), ROS and MDA level (**B**), and LPO level (**C**) of ZFL after being treated with 3 μM RSL3 for 6 h (*n* = 6). Distribution of ROS (green fluorescence) in ZFL in each incubation pattern (**D**). Distribution images of ROS were taken using an inverted fluorescent microscope (Leica DMi8, Germany) in the FITC green fluorescent channel with a magnification of 10×. Error bars represent the standard error of each group, and means with different superscript letters are significantly different (*p* < 0.05),while the same letter means no statistical difference between two groups. In figure B, uppercase letters (A, B, C, D) and lowercase letters (a, b, c, d) indicate the statistical difference of ROS level and MDA level in each group, respectively.

Since RSL3 is a specific inhibitor of GPx4, detecting the activity of total GPx activity in ZFL reveals the effect of 1,25(OH)_2_D_3_ on the overall intracellular antioxidant levels and not limited to lipid peroxidation. The activity of total GPx in ZFL is shown in Figure 3A. Compared with the 0-0 group, the activity of total GPx in the 0-R group was significantly decreased (*p* < 0.05), indicating that RSL3 could deplete intracellular GPx and reduce its activity. Compared with the 0-R group, the 0-V-R, V-R and 0-(V+R) incubation patterns all increased the activity of total GPx to different degrees (*p* < 0.05). Among them, the 0-V-R group and V-R group had the strongest effect on the activity of increasing total GPx. No significant difference in total GPx was found between the 0-V-R group and V-R group (*p* > 0.05). 

Since ROS and MDA are one of the substrates and products of lipid peroxidation, respectively, the effects of different incubation patterns of 1,25(OH)_2_D_3_ on ROS and MDA levels of ZFL treated with 3 μM RSL3 for 6 h are shown in Figure 3B. Figure 3C shows the level of LPO within the ZFL. Figure 3D shows the fluorescent intensity of ROS of ZFL in different incubation patterns. The results showed that RSL3 stimulation caused ZFL to produce a large amount of ROS, LPO, and MDA (*p* < 0.05), while 1,25(OH)_2_D_3_ incubation significantly reduced the production of ROS, LPO, and MDA in RSL3-stimulated ZFL. As for the effects of different 1,25(OH)_2_D_3_ incubation patterns, the 0-(V+R) group exhibited the lowest level of ROS, 0-V-R was the next, and V-R showed the highest level of ROS among these three groups (*p* < 0.05). The levels of LPO in the V-R group were significantly reduced when compared to the 0-V-R and 0-(V+R) groups (*p* < 0.05). For MDA, the V-R group had the strongest ability to reduce the MDA level (*p* < 0.05), while 1,25(OH)_2_D_3_ preincubation for 6 h (0-V-R group) and co-incubation for 6 h (0-(V+R) group) had no difference in reducing the MDA level in ZFL (*p* > 0.05). 

The above results revealed that 1,25(OH)_2_D_3_ incubation, whether it was preincubation for 6 h or 72 h or co-incubation with RSL3 for 6 h, enhanced the intracellular total GPx activity to a certain extent, and reduced ROS, LPO, and MDA levels, thus protecting ZFL from damage caused by RSL3.

### 2.4. Effect of 1,25(OH)_2_D_3_ on Iron Ion Level of ZFL

**Figure 4:**
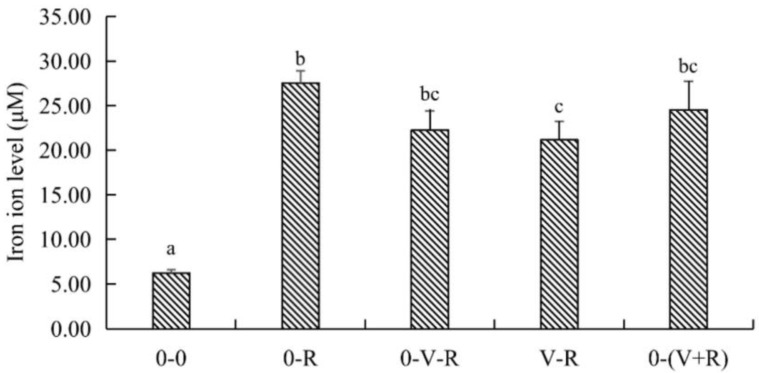
Effects of different incubation patterns of 1,25(OH)_2_D_3_ on iron ion level of ZFL after being treated with 3 μM RSL3 for 6 h (*n* = 6). Error bars represent the standard error of each group, and means with different superscript letters (a, b, c) are significantly different (*p* < 0.05),while the same letter means no statistical difference between two groups.

The effect of different incubation patterns of 1,25(OH)_2_D_3_ on intracellular iron level of ZFL treated with 3 μM RSL3 for 6 h is shown in Figure 4. Compared with the 0-0 group, the level of iron in the 0-R group was significantly increased (*p* < 0.05), implying that RSL3 treatment could stimulate intracellular iron accumulation. Compared with the 0-R group, the iron ion level in the V-R group was significantly decreased (*p* < 0.05), while the iron ion levels in the 0-V-R and 0-(V+R) groups were not significantly different from that in the 0-R group (*p* > 0.05). The above results showed that 1,25(OH)_2_D_3_ preincubation for 72 h could inhibit the intracellular iron aggregation of ZFL stimulated by RSL3. 

### 2.5. Screening and Functional Enrichment of DEGs

**Figure 5:**
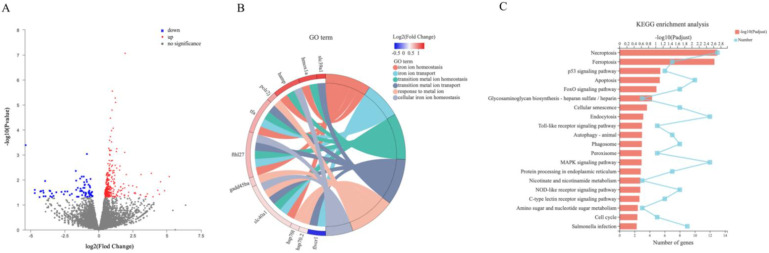
Volcano plots of differently expressed genes (DEGs) (**A**), chord diagram of signal pathways related to iron ion transport and metal ion transport (GO term) (**B**), and Top-20 pathways with the most significant enrichment by KEGG (**C**). All the DEGs were screened according to |log2(Fold change)| ≥ 1 and *p*-adjust < 0.05. In the volcano plots, the red dots represent upregulated genes, blue dots represent downregulated genes, and grey dots represent genes that do not meet the above screening conditions. Different colors in the chord graphs represent different signaling pathways, and log2(Fold change) value is shown in color gradient. In picture (**C**), the red bar represents the value of −log10(*p*-adjust) and the light blue line represents the number of genes in the corresponding signaling pathway.

A total of 37.89 Gb clean data was obtained from NGS analysis, and 22,858 expressed genes were detected. After being compared with the reference database (Danio rerio, http://asia.ensembl.org/Danio_rerio/Info/Index), 21,586 known genes were found. According to *p*-adjust < 0.05 and |log2(Fold Change)| ≥ 1543 DEGs were screened, including 310 upregulated genes and 233 downregulated genes, which were displayed together in the volcano map (Figure 5A). The 543 DEGs were functionally enriched in GO and KEGG websites. Figure 5B shows the GO enrichment of six signaling pathways related to iron and metal ion transport and the corresponding genes, among which the hepcidin gene hamp appeared simultaneously in three signaling pathways related to iron ion transport. Figure 5C shows the most significant Top-20 signaling pathways and the number of corresponding genes in KEGG enrichment, including the ‘ferroptosis’ signaling pathway. The above results indicated that 1,25(OH)_2_D_3_ preincubation for 72 h significantly affected the iron ion transport and ferroptosis signaling pathway.

### 2.6. Effect of 1,25(OH)_2_D_3_ on Gene Expression Related to Keap1–Nrf2–GPx4 and NF-κB–hepcidin Axis

**Figure 6:**
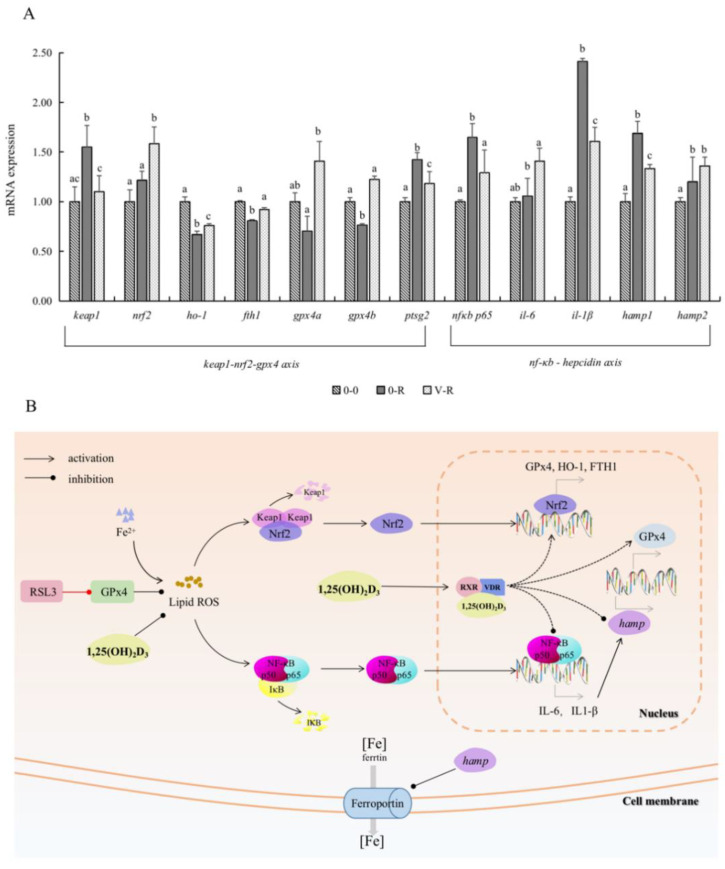
Effects of 1,25(OH)_2_D_3_ on the gene expression related to the Keap1–Nrf2–GPx4 and NF-κB–hepcidin axis (**A**) and visualization of Keap1–Nrf2–GPx4 axis and NF-κB–hepcidin axis (**B**). ZFL were collected for qPCR after being preincubated with 200 pM 1,25(OH)_2_D_3_ for 72 h and then treated with 3 μM RSL3 for 6 h. Values are presented as means (*n* = 6) and error bars represent the standard error of each group. Means with different superscript letters (a, b, c) are significantly different (*p* < 0.05), while the same letter means no statistical difference between two groups. Abbreviations in (**B**): RSL3, specific inhibitor of GPx4; GPx4, glutathione peroxidase 4; ROS, reactive oxygen species; Keap1, Kelch-like ECH-associated protein-1; Nrf2, nuclear factor erythroid 2-related factor 2; NF-κB p65, nuclear factor kappa B p65; IκB, inhibitor of nuclear factor kappa-B; IL-6, interleukin 6; IL-1β, interleukin 1β; RXR, retinoid X receptor; VDR, vitamin D receptor; hamp, hepcidin antimicrobial peptide gene; HO-1, heme oxygenase-1; FTH1, ferritin heavy chain 1.

Figure 6A shows the expression of Keap1–Nrf2–GPx4 and NF-κB–hepcidin axis-related genes after ZFL were treated with 3 μM RSL3 for 6 h, with 72 h preincubation of 1,25(OH)_2_D_3_. In the Keap1–Nrf2–Gpx4 axis, the expression of *keap1* and *ptgs2* in the 0-R group was significantly upregulated compared with that in the 0-0 group (*p* < 0.05), and the expression of *nrf2*, *ho-1*, *fth1*, *gpx4a*, and *gpx4b* were all downregulated to a certain extent. These results indicated that RSL3 could promote the expression of *keap1* and *ptgs2* genes and inhibit the expression of the antioxidant genes *ho-1*, *gpx4*, and iron-storage-related gene *fth1* downstream of Nrf2. Compared with the 0-R group, the expression of *keap1* and *ptgs2* genes in the V-R group was decreased (*p* < 0.05), while the expression of *nrf2*, *ho-1*, *fth1*, *gpx4a*, and *gpx4b* were significantly upregulated (*p* < 0.05). The above results revealed that 1,25(OH)_2_D_3_ preincubation for 72 h could inhibit the expression of the *keap1* gene, thereby activating *nrf2* and promoting the expression of downstream antioxidant genes *ho-1*, *gpx4*, and iron-storage-related gene *fth1*, inhibiting the expression of ferroptosis-sensitive factor *ptgs2*. 

On the other hand, the expression levels of NF-κB signal pathway key factor NF-κB p65 and inflammatory factors IL-6 and IL-1β in the 0-R group were significantly upregulated compared with the 0-0 group (*p* < 0.05), indicating that RSL3 activated the NF-κB signal pathway and released inflammatory factors. In addition, *il-6* and *il1-β* are precursors of *hamp*; therefore, the expression levels of *hamp1* and *hamp2* in the 0-R group showed an increasing trend (*p* < 0.05). Compared with the 0-R group, the expression levels of *nf-κb p65*, *il-6*, and *il1-β* in the V-R group were significantly decreased (*p* < 0.05), denoting that 1,25(OH)_2_D_3_ preincubation for 72 h could inhibit the NF-κB signal pathway. The expression level of *hamp1* gene was downregulated in the V-R group compared with the 0-R group (*p* < 0.05), while the expression level of *hamp2* gene was upregulated in the V-R group, but there was no statistical difference (*p* > 0.05).

Based on the above results, Figure 6B shows a simulated diagram of the regulatory mechanism of 1,25(OH)_2_D_3_ on ferroptosis through the Keap1–Nrf2–GPx4 and NF-κB–hepcidin axis. Keap1 is an inhibitor of Nrf2. In the resting state, the Keap1–Nrf2 protein complex is inactivated. After being treated with exogenous RSL3, a large amount of GPx4 was depleted, which led to a sharp increase in ROS level in ZFL, promoted the expression of *keap1*, and inhibited the Nrf2–GPx4 axis. The NF-κB signal pathway was activated by the increasing ROS, and the expression of *il-6* and *il1-β* increased accordingly, which resulted in the increase in *hamp* gene expression. Ferroprotin function was inhibited by the enhanced expression of *hamp* and, thus, caused a large aggregation of intracellular Fe^2+^ and lipid peroxidation reactions, which eventually caused ferroptosis. 1,25(OH)_2_D_3_ regulated the homeostasis of iron ions in zebrafish liver cells in both direct and indirect ways. On the one hand, after exogenous supplementation of 1,25(OH)_2_D_3_, 1,25(OH)_2_D_3_ directly worked on the expression of *gpx4* and hamp genes to regulate the function of ferroportin. On the other hand, exogenous 1,25(OH)_2_D_3_ reduced the expression of *keap1*, thus activating the Nrf2–GPx4 axis, increasing the expression of *ho-1*, *fth1*, and *gpx4* gene, decreasing the expression of *ptgs2*, and enhancing the activity of antioxidant enzyme GPx4. In addition, since the NF-κB signal pathway was inhibited by exogenous 1,25(OH)_2_D_3_, the expression of hamp gene in the nucleus was also inhibited due to the inhibition of its precursors, i.e., *il-6* and *il1-β* in the NF-κB signal pathway. Therefore, ferroprotin could normally play the function of transporting iron ions so that intracellular iron ions could be transported out of the cell, reducing the occurrence of lipid peroxidation, and thereby reducing RSL3-induced ferroptosis of cells.

## 3. Discussion

Ferroptosis occurrence is related to the metabolism of iron, glutathione, ROS, and LPO. Extracellular Fe^3+^ is transferred to the cell via transferrin and reduced to Fe^2+^, which reacts with excess H_2_O_2_ in the cell to produce a large amount of ROS, promoting the production of intracellular LPO, and thus triggering ferroptosis [2]. In humans, iron overload leads to hereditary hemochromatosis (HH), increased production of ROS in the body [31], and iron-overload anemia [32]. Due to their special living environment, fish are more susceptible than humans and mammals to environmental changes, such as iron overload in water or invasion by pathogens. In fish, iron overload in the environment causes the immune system of Atlantic salmon to be damaged, showing a high susceptibility to *Piscirickettsia salmonis* [33]. Jiang et al. [34] reported that iron overload in the environment would lead to a decrease in the systemic iron content and oxidative stress of zebrafish embryos. In addition, Yang et al. [35] found that *E. coli* can induce ferroptosis of grass carp erythrocytes. As mentioned above, VD has been shown to be a regulator of antioxidant capability and iron metabolism in recent years; therefore, studying the relationship between VD and ferroptosis of fish cells has important significance to promote the healthy development of fisheries. 

In the current study, the addition of exogenous ferroptosis activator RSL3 caused ferroptosis in zebrafish liver cells, and significant changes in the internal structure of the cells were found, such as mitochondrial volume reduction, crestal shrinkage, and outer membrane dissolution, which is consistent with the morphological characteristics of previously reported ferroptosis cells [2]. In terms of physiological indicators, during the process of ferroptosis, the content and activity of total GPx in ferroptosis zebrafish liver cells decreased, and a large amount of ROS and LPO production was observed, and MDA content, a product of lipid peroxidation, increased accordingly, as well as a large amount of intracellular iron aggregation. These results were the typical characteristics of ferroptosis, as reported previously [3,4]. The current study found that no matter what the incubation pattern was, the addition of 1,25(OH)_2_D_3_ improved the survival rate of ZFL, alleviated the pathological morphological changes of mitochondria, enhanced the intracellular total GPx activity, reduced the intracellular ROS and MDA production, and thereby inhibited ferroptosis of ZFL induced by RSL3. The pattern of 1,25(OH)_2_D_3_ preincubation for 72 h (V-R group) achieved the best inhibitory effect on ferroptosis. These results found in zebrafish liver cells were similar to previous studies done in humans and mammals. Hu et al. [29] reported that VDR activators attenuated cisplatin-induced ferroptosis in mouse kidneys by reducing lipid peroxidation and MDA production, and reversing GPx4 downregulation. Cui et al. [36] found that VDR activator treatment could affect the activity of NADPH oxidase in the brain injury model of mice, thereby inhibiting apoptosis. VDR can also alleviate abnormal lipid metabolism in mice [37]. In human studies, VDR overexpression and VD treatment have been shown to reduce apoptosis and mitochondrial damage in patients with gestational diabetes [38]. Moreover, VD treatment can prevent the oxidative stress of renal tubule cells caused by high glucose, improve the activity of SOD, and reduce the production of MDA [39]. In fish, dietary VD can increase the activity of serum peroxidase in sea bream (*Sparus aurata L.*) [20] and European sea bass (*Dicentrarchus labrax L.*) [40]. In addition, previous studies in our lab have also found that dietary VD_3_ or cell culture medium supplemented with 1,25(OH)_2_D_3_ can improve the antioxidant capacity of yellow catfish or head kidney macrophages and reduce the production of ROS [23,24,25]. All this evidence underscores the role of VD_3_ in resistance to lipid peroxidation and ferroptosis in animals.

The current study examined the expression of genes related to the Keap1–Nrf2–GPx4 axis, a key regulatory pathway in ferroptosis, as well as hepcidin gene (*hamp*), which is a critical factor in iron homeostasis. The Nrf2-mediated antioxidant pathway has been considered to be the key to activate the cell defense system and resist oxidative damage [41]. Previous studies have shown that the state of Kelch-like ECH-associated protein-1 (Keap1) cysteine residue changes in the presence of exogenous stimuli, leading to the nuclear translocation of Nrf2, which is an important antioxidant factor. Nrf2 then binds to antioxidant responsive element (ARE) in the nucleus to promote the expression of antioxidant enzyme genes (such as GPx4, heme oxygenase-1 (HO-1)) [42]; meanwhile, Nrf2 can also regulate the expression of ferritin heavy chain 1 (FTH1), a gene associated with iron ion storage [43]. Previous studies demonstrated that inhibition of Nrf2 abolished the protective effect of tanshinone IIA on human coronary artery endothelial cell ferroptosis and that Nrf2 knockdown inhibited the anti-ferroptosis effect of melatonin on lung epithelial cells, suggesting a role of Nrf2 in inhibiting ferroptosis [44]. As previously mentioned, GPx4 is known to regulate inflammatory responses and apoptosis in humans and mammals, in addition to its antioxidant capacity [45]. According to Thisse et al. [46], zebrafish have two GPx4 genes, i.e., *gpx4a* and *gpx4b*, but research on the function of GPx4 in fish is incomplete. In this study, it was found that the expression of *keap1* is upregulated and *nrf2*, *ho-1*, *fth1, gpx4a*, and *gpx4b* were downregulated in ZFL after RSL3 stimulation, while 1,25(OH)_2_D_3_ treatment could inhibit the expression of *keap1* and upregulate the expression of *nrf2*, *ho-1*, *fth1*, *gpx4a*, and *gpx4b*. In addition, PTGS2 is a sensitive factor for ferroptosis caused by GPx4 inactivation. The current study found that the expression of *ptgs2* gene in ZFL increased significantly after RSL3 stimulation, and, after 1,25(OH)_2_D_3_ treatment, the expression of *ptgs2* gene in ZFL was a downward trend. Combined with the results of physiological indicators, we made the inference that 1,25(OH)_2_D_3_ may reverse the RSL3-induced downregulation of *ho-1*, *fth1*, *gpx4a*, and *gpx4b* genes through the Keap1–Nrf2–GPx4 axis and increase the activity of antioxidant enzyme GPx4, thereby inhibiting ROS and LPO production and reducing MDA content in ZFL. Actually, our inference was confirmed by the research carried out by El-Magd and Eraky [47], in which vitamin D played an antioxidant role by regulating the Nrf2/HO-1 signaling pathway when studying rat models of liver and kidney injury. Abbaszadeh et al. [48] also found that VD_3_ regulated oxidative stress and inflammation induced by Pb poisoning in mice through Nrf2 and NF-κB signaling pathways. Moreover, our results agreed with the findings in mice, which showed that VDR depletion significantly reduced *gpx4* expression, while *gpx4* expression was upregulated with the use of VDR activator [29]. However, the direct interaction between VD and GPx4 has not been well documented. 

Hepcidin has both antimicrobial and inhibitory functions against membrane ferroportin, and is an important factor regulating iron metabolism and iron balance in animals [49,50]. In fish, hamp has two isoforms, called *hamp1* and *hamp2*; among them, *hamp1* is a single copy, highly homologous to mammals, and is mainly involved in regulation of iron metabolism and maintenance of iron homeostasis, while *hamp2* has multiple copies and plays an antimicrobial role, which may indicate that hepcidin plays a unique role in immune responses to different pathogens [51,52]. Susu et al. [53] found that 1,25(OH)_2_D_3_ reduced the level of IL-6 and IL-1β, indicating that VD may directly reduce the release of pre-hepcidin cytokines and inhibit the production of hepcidin. The same results were obtained in the current study, which showed that 1,25(OH)_2_D_3_ preincubation could inhibit NF-κB signal pathway activation in ZFL after RSL3 stimulation and reduce IL-6 and IL-1β production. Meanwhile, the expression of *hamp1* and *hamp2* was significantly upregulated in ZFL with ferroptosis induced by RSL3 stimulation without 1,25(OH)_2_D_3_. However, after preincubation with 200 pM 1,25(OH)_2_D_3_ for 72 h, the expression of *hamp1* was downregulated, while *hamp2* showed an upregulation trend. The difference between *hamp1* and *hamp2* expression may be related to the function of two different isoforms of *hamp*, as mentioned above. In the RSL3-induced ferroptosis model of ZFL, 1,25(OH)_2_D_3_ mainly affected the expression of *hamp1* and maintained iron homeostasis of ZFL. Therefore, in this study, in addition to regulating the expression of Keap1–Nrf2–GPx4 axis, 1,25(OH)_2_D_3_ can also reduce the production of *il-6* and *il-1β* by inhibiting the NF-κB signal pathway, thereby reducing the expression of *hamp1*, weakening the inhibitory effect on membrane ferroportin, thus accelerating the output of iron ions in ZFL, reducing intracellular iron aggregation and decreasing the occurrence of ferroptosis. Studies on the effect of VD on hepcidin levels in humans have been reported. Smith et al. [54] found that a high dose of VD_3_ could significantly reduce plasma hepcidin concentrations in healthy adults within one week after administration, but plasma proinflammatory factors and ferritin concentrations did not change. This suggests that VD_3_ may also play an iron regulatory role independent of changes in proinflammatory factors. In addition, the results of NGS in the current study also confirmed that 1,25(OH)_2_D_3_ could influence iron ion transport and ferroptosis signaling pathway to regulate RSL3-induced ferroptosis of ZFL. Bacchetta et al. [28] described the regulatory mechanism of VD on the hepcidin–ferroportin–iron regulatory axis in detail: in the absence of VD, increased hepcidin synthesis in liver cells may increase intracellular and circulating hepcidin concentration and decrease membrane expression of ferroportin in these cells. Subsequently, iron output is inhibited, leading to iron accumulation in the cell. Under the condition of sufficient VD, reduced transcription of *hamp* may lead to decreased intracellular and circulatory hepcidin concentration and increased membrane expression of ferroportin, thus increasing iron output and resulting in decreased intracellular iron ion concentration. In addition, Tanaka and Teitelbaum [55] also found that the expression of the transferrin receptor regulating iron ion absorption in bone-marrow-derived macrophages decreased by at least 30% in a dose-dependent manner after 1,25(OH)_2_D_3_ culture, indicating that 1,25(OH)_2_D_3_ not only can promote cell export of iron ions under ferroptosis, but also inhibit cell absorption of iron ions, thus alleviating intracellular iron ion aggregation and reducing the negative effects of ferroptosis on cells. As far as we know, there is only one piece of evidence for the effect of VD on hepcidin level in fish. Li et al. [56] found that dietary supplementation of 0, 250, 500, 1000, 2000, and 4000 IU/kg VD_3_ increased the expression of hamp in hepatopancreas, spleen, head kidney, and hindgut of Monopterus albus (*Asian swamp eel*) at first, and then decreased. However, this study in fish only showed the symptom and did not explain the action mechanism of VD_3_. Further research is needed to deepen our understanding on the mechanism by which VD regulates ferroptosis in fish.

In conclusion, this study confirmed the protective effect of 1,25(OH)_2_D_3_ on RSL3-induced ferroptosis in zebrafish liver cells, and reported for the first time that 1,25(OH)_2_D_3_ inhibits ferroptosis in fish cells by regulating the Keap1–Nrf2–GPx4 axis and NF-κB–hepcidin axis. This study provides new evidence for the function of VD in fish and the regulatory mechanism of ferroptosis in fish cells, as well as a better understanding of vitamin D benefit in aquatic feed industry fish.

## 4. Materials and Methods

### 4.1. Cell Culture

Zebrafish liver cell line (ZFL) was kindly provided by the China Zebrafish Resource Center (CZRC catalog ID: Cell2, http://zfish.cn/Products/ProductDetail.aspx?CZRCID=Cell2, access date: 13 October 2020) and stored in the Preservation and Research Center for Aquatic Animal Cells, Huazhong Agriculture University. ZFL grew in a mixed medium consisting of 48% Leibovitz’s L-15, 15% Ham’s F-12, 32% Dulbecco’s modified Eagle medium (Gibco, Carlsbad, CA, USA), 4% fetal bovine serum (FBS) (Gibco, Carlsbad, CA, USA), and 1% antibiotics (50 U/mL anti-penicillin and 50 μg/mL anti-streptomycin) (Gibco, Carlsbad, CA, USA). The cells were cultured in a cell culture flask (base area: 25 cm^2^) in a conventionally humidified incubator at 28 ℃, 5% CO_2_, and 4 mL fresh medium was replaced at intervals of one day. When the monolayer cell density was greater than 90%, the cells were digested with 0.25% trypsin and cell passage cultivation was carried out.

### 4.2. 1,25(OH)_2_D_3_ Incubation and Ferroptosis Inducer (RSL3) Treatment

The pre-experimental results showed that the cell viability was the strongest when ZFL was cultured for 72 h. Supplementation of 200 pM 1,25(OH)_2_D_3_ (Selleck Chemicals, Houston, TX, USA) in the cell culture medium had no effect on the viability of ZFL for 72 h and the lethal concentration for 50% (LC50) of ZFL stimulated by RSL3 (Glpbio, Montclair, CA, USA) for 6 h was 3 μM (Table S1, Figure S1, see Supplementary Materials).

Before the experiment, ZFL with a monolayer cell density of over 90% was digested into suspension with 0.25% trypsin. The obtained ZFL suspensions were first precultured for 24 h and the non-adhesive cells were removed for later use. In order to explore the protective effect of 1,25(OH)_2_D_3_ on RSL3-induced ZFL ferroptosis by different preincubation periods (6 h or 72 h) and different incubation patterns (preincubation or co-incubation), the above adherent ZFL were categorized as follows:

0-0 (control group): First 1,25(OH)_2_D_3_-free for 72 h, then RSL3-free for 6 h;

0-R (positive control group): First 1,25(OH)_2_D_3_-free for 72 h, then 3 μM RSL3 for 6 h;

0-V-R (1,25(OH)_2_D_3_ preincubated for 6 h): First 1,25(OH)_2_D_3_-free for 66 h, then 200 pM 1,25(OH)_2_D_3_ for 6 h, and then 3 μM RSL3 for 6 h;

V-R (1,25(OH)_2_D_3_ preincubated for 72 h): First 200 pM 1,25(OH)_2_D_3_ for 72 h, then 3 μM RSL3 for 6 h; 

0-(V+R) (1,25(OH)_2_D_3_ and RSL3 co-incubated for 6 h): First 1,25(OH)_2_D_3_-free for 72 h, then 200 pM 1,25(OH)_2_D_3_ + 3 μM RSL3 for 6 h.

The culture conditions were consistent with those described in Section 4.1.

### 4.3. Morphological Observation and Survival Detection of ZFL

For the purpose of observing the morphology of the ZFL, the cells treated in Section 4.2 were collected and then fixed with a transmission electron microscopy fixation solution. The mitochondrial morphology of the ZFL was observed on the transmission electron microscope (TEM, Hitachi TEM system, Tokyo, Japan). Cell survival rate was accomplished by the Cell Counting Kit-8 (CCK-8) (Dojindo, Kumamoto Ken, Japan), and the experimental methods followed the instructions. In a few words, the ZFL suspension was seeded in a 96-well plate (6 × 10^4^ cells/well) with the same grouping and treatment as in Section 4.2. After treatment, 10 μL CCK-8 was added to each well and incubated for 3 h in the incubator, and then detected with Infinite M200 NanoQuant (Tecan, Männedorf, Switzerland) at wavelength 450 nm.

### 4.4. Reactive Oxygen Species (ROS)and Lipid Peroxidation (LPO) Detection

The detection of reactive oxygen species in ZFL was accomplished by the Reactive Oxygen Species Assay kit (Fanbo Biochemical, Beijing, China). The DCFH-DA fluorescent probe was provided in the kit. The ZFL suspension was seeded in a 96-well plate (6 × 10^4^ cells/well) with the same grouping and treatment as in Section 4.2. After treatment, the DCFH-DA fluorescent probe was installed in situ. Simply put, the medium in the 96-well plate was first discarded. Then, 100 μL of diluted DCFH-DA fluorescent probe was added to each well after the DCFH-DA was diluted with serum- and antibiotic-free medium at a ratio of 1:1000. Subsequently, the 96-well plate was incubated in an incubator for 20 min. After the incubation, the DCFH-DA fluorescent probe in the 96-well plate was discarded and the 96-well plate was washed twice with PBS, whereafter, the fluorescence values were detected by SpectraMax^®^i3x (Molecular Devices, Sunnyvale, CA, USA) (excitation/emission: 488/525 nm). The intensity of the fluorescence indicated the level of ROS in the ZFL. Meanwhile, ZFL were observed and photographed with an inverted fluorescence microscope (Leica DMi8, Wetzlar, Germany). Light avoidance was required for installing the probes and during the testing process.

The lipid peroxide fluorescent probe Liperfluo (DOJINDO, Kumamoto Ken, Japan) was used to detect the content of LPO within the ZFL. The ZFL suspension was seeded in a 96-well plate (6 × 10^4^ cells/well) with the same grouping and treatment as in Section 4.2. After treatment, the medium was removed; after cells were washed twice with PBS, 100 μL 1 μmol/L Liperfluo fluorescence probe was added to each well and 96-well plates were incubated in the incubator for 30 min. After the incubation, the Liperfluo fluorescent probe in the 96-well plate was discarded and the 96-well plate was washed twice with PBS, whereafter, the fluorescence values were detected by SpectraMax^®^i3x (Molecular Devices, Sunnyvale, CA, USA) (excitation/emission: 488/525 nm). Light avoidance was required for installing the probes and during the testing process.

### 4.5. Malondialdehyde (MDA), Iron Ion Level, and Total Glutathione Peroxidase (GPx) Activity Detection

The ZFL suspension was seeded in a 24-well plate (2.4 × 10^5^ cells/well) with the same grouping and treatment as in Section 4.2. 

A total protein assay kit (BCA method) and Cell Malondialdehyde (MDA) assay kit (Colorimetric method) (Jiancheng Bioengineering Institute, Nanjing, China) were used to detect the protein concentration and MDA content in ZFL samples, respectively. Infinite M200 Nanoquant (Tecan, Männedorf, Switzerland) was used to detect the absorption value at the wavelength of 562 nm and 532 nm, respectively.

Intracellular Iron ColorimesRic Assay Kit (Applygen, Beijing, China) was used to detect the level of iron ions in the ZFL. Standard samples (from the kit) and ZFL samples were treated according to the method described in the kit instructions. Infinite M200 Nanoquant (Tecan, Männedorf, Switzerland) was employed to detect the absorption value at the wavelength of 550 nm. The content of iron ions in the ZFL samples was calculated according to the standard curve.

Standard samples (from the kit) and ZFL samples were treated according to the method described in the Glutathione Peroxidase Assay Kit II (Applygen, Beijing, China) instructions. Infinite M200 Nanoquant (Tecan, Männedorf, Switzerland) was used to detect the absorption value at the wavelength of 340 nm. The NADPH standard curve was drawn to calculate the amount of NADPH in each ZFL sample. Total GPx activity was calculated as:


Total GPx activity (mU) = NADPH amount (nmol) ∗ 2/Reaction time (min)

### 4.6. Next-Generation Sequencing (NGS) Analysis of ZFL in the 0-R and V-R Group

The results of the above experiments showed that the V-R group has the most effective inhibitory effect on the ferroptosis of ZFL induced by RSL3, so the 0-R and V-R groups were selected for NGS analysis in the current experiment. Illumina high-throughput sequencing is mainly divided into four steps: library preparation, cluster generation, sequencing, and data analysis (Majorbio CO., LTD, Shanghai, China). For specific principles and methods, please refer to Van Dijk et al. [57] and Dillies et al. [58]. The raw data obtained by NGS were first base-evaluated on the fastx_toolkit_0.0.14 software, then the quality of the raw data was evaluated on the SeqPrep (https://github.com/jstjohn/SeqPrep) and Sickle (https://github.com/najoshi/sickle) software to obtain high-quality clean data. Subsequently, TopHat2 (http://ccb.jhu.edu/software/tophat/index.shtml) and HISAT2 (http://ccb.jhu.edu/software/hisat2/index.shtml) software were used for sequence matching analysis. Based on the selected reference genome sequence, the assembled transcripts were compared with the reference transcript to obtain the difference information of NGS using Cufflinks (http://cole-trapnell-lab.github.io/cufflinks/) software. Clean data were compared in NCBI_NR (NCBI nonredundant protein library) (ftp://ftp.ncbi.nlm.nih.gov/blast/db/, access date: 25 July 2021) to obtain gene information and annotated gene functions. Fragments per kilobases per million reads (FPKM) were used to calculate gene expression levels. Then, the differently expressed genes (DEGs) were analyzed in the DESeq2 software according to *p*-adjust < 0.05 and |log2(Fold Change)| ≥ 1. The obtained DEGs were respectively enriched in signal pathways on GO (Gene Ontology, http://www.geneontology.org, access date: 30 July 2021) and KEGG (Kyoto Encyclopedia of Genes and Genomes, http://www.genome.jp/kegg/, access date: 30 July 2021) websites.

### 4.7. Detection of Gene Expression Related to Keap1–Nrf2–GPx4 and NF-κB–hepcidin Axis

The ZFL suspension was seeded in cell culture flasks (base area: 25 cm^2^, 1.8 × 10^6^ cells/flask) with the same grouping and treatment as in Section 4.2. After treatment, the medium was discarded and ZFL were washed twice with PBS; then, 1 mL TRIzol reagent (Invitrogen, Carlsbad, CA, USA) was added to each cell culture flask to lyse the ZFL samples for extraction of the total RNA. Subsequently, Prime Script^TM^ RT reagent kit with gDNA Eraser (Perfect Real Time) (Takara, Tokyo, Japan) was used for the synthesis of cDNA, the obtained cDNA was diluted 5 times with DEPC water for use. The primer sequences of Keap1–Nrf2–GPx4 and NF-κB–hepcidin axis-related genes are shown in the Table S2, and *β-actin* was used as a housekeeping gene due to the high stability of mRNA levels in all the treatments. qPCR was completed using Hieff^TM^ qPCR SYBR Green Master Mix (Yeasen, Shanghai, China) kit on the QuantStudio^TM^ 6 Flex real-time PCR System (Thermo Fisher Scientific, Waltham, MA, USA); amplification efficiency of all genes was greater than 95%. The 2^−ΔΔCt^ method was used to analyze the raw gene expression data [59].

### 4.8. Statistical Analysis

All the data obtained were represented as mean ± standard error (SE). Shapiro–Wilk’s test was used to perform a normal distribution test of the data, and Levene’s test was used as a homogeneity of variance test. One-way ANOVA was adopted for detecting the difference among different treatments, followed by least significant difference (LSD) for inter-group multiple comparisons, and *p*-value < 0.05 represented significant differences.

## Data Availability

The original data of NGS have been uploaded to NCBI (SRA accession number: PRJNA772154). The original contributions presented in the study are included in the article. Further inquiries can be directed to the corresponding author.

## Supplementary Materials

The following are available online at www.mdpi.com/1422-0067/222/11/1334/s1, Table S1: Survival rate of ZFL challenged with different doses of RSL3 for 6 h. Table S2: Nucleotide sequences of qPCR primers used in this study. Figure S1: Prediction of the lethal concentration 50% (LC50) of ZFL challenged with RSL3 for 6 h.
