# Supplementary material for: 1,25(OH)2D3 Inhibited Ferroptosis in Zebrafish Liver Cells (ZFL) by Regulating Keap1-Nrf2-GPx4 and NF-κB-hepcidin Axis
Source: Int J Mol Sci. 2021 Oct 20;22(21):11334. doi: 10.3390/ijms222111334 (PMC8583391; doi:10.3390/ijms222111334)
Supplement: Supplementary file 1 [file ijms-22-11334-s001.zip › Supplementary materials.pdf]

Supplementary materials

Table S1. Survival rate of ZFL challenged with different doses of RSL3 for 6 h

Dose of RSL3	OD_{450nm}						mean	Survival rate[a]
Blank	0.175	0.164	0.179				0.173	
Ctrl (0 μM)	0.649	0.635	0.654	0.652	0.69	0.663	0.657	100%
1.0 μM	0.47	0.512	0.458	0.447	0.454	0.492	0.472	61.78%
2.5 μM	0.442	0.439	0.425	0.472	0.429	0.413	0.437	54.55%
5.0 μM	0.385	0.379	0.394	0.397	0.364	0.392	0.385	43.80%
7.5 μM	0.353	0.357	0.374	0.349	0.342	0.331	0.351	36.78%
10.0 μM	0.346	0.332	0.343	0.345	0.352	0.329	0.341	34.71%

[a] Survival rate was calculated as: $[(OD_{treatment\ group}-OD_{blank})/(OD_{Ctrl}-OD_{blank})]\times100\%$

Confidence Limits

	Probability	95% Confidence Limits for Dose_RSL3			95% Confidence Limits for log(Dose_RSL3)[a]		
		Estimate	Lower Bound	Upper Bound	Estimate	Lower Bound	Upper Bound
LOGIT	.010	.000	.000	.006	-3.458	-6.539	-2.230
	.020	.001	.000	.016	-2.857	-5.484	-1.809
	.030	.003	.000	.028	-2.502	-4.860	-1.561
	.040	.006	.000	.041	-2.248	-4.413	-1.382
	.050	.009	.000	.057	-2.048	-4.063	-1.242
	.060	.013	.000	.075	-1.883	-3.774	-1.126
	.070	.018	.000	.094	-1.743	-3.527	-1.027
	.080	.024	.000	.115	-1.619	-3.311	-.940
	.090	.031	.001	.137	-1.510	-3.118	-.863
	.100	.039	.001	.161	-1.410	-2.944	-.793
	.150	.097	.006	.306	-1.015	-2.252	-.514
	.200	.192	.019	.498	-.718	-1.733	-.302
	.250	.337	.050	.748	-.472	-1.305	-.126
	.300	.553	.117	1.070	-.258	-.934	.030
	.350	.866	.252	1.493	-.063	-.599	.174
	.400	1.317	.512	2.062	.120	-.291	.314
	.450	1.971	.988	2.874	.295	-.005	.458
	.500	2.924	1.796	4.167	.466	.254	.620
	.550	4.338	2.992	6.598	.637	.476	.819
	.600	6.488	4.532	11.719	.812	.656	1.069
	.650	9.874	6.511	22.892	.994	.814	1.360
	.700	15.466	9.258	48.517	1.189	.967	1.686
	.750	25.349	13.390	112.991	1.404	1.127	2.053
	.800	44.627	20.207	300.654	1.650	1.305	2.478
	.850	88.511	33.006	990.711	1.947	1.519	2.996
	.900	219.789	62.936	4858.771	2.342	1.799	3.687
	.910	276.313	73.985	7254.136	2.441	1.869	3.861
	.920	355.877	88.454	11301.428	2.551	1.947	4.053
	.930	472.656	108.048	18585.008	2.675	2.034	4.269
	.940	653.578	135.752	32808.520	2.815	2.133	4.516
	.950	955.006	177.268	63817.756	2.980	2.249	4.805
	.960	1511.735	244.810	142895.622	3.179	2.389	5.155
	.970	2716.176	369.420	399780.330	3.434	2.568	5.602
	.980	6150.597	655.417	1679888.84	3.789	2.817	6.225
	.990	24514.434	1727.085	19075142.9	4.389	3.237	7.280

a. Logarithm base = 10.

Figure S1. Prediction of the lethal concentration 50% (LC50) of ZFL challenged with RSL3 for 6 h. (Software: IBM SPSS Statistics, version 25.0)

Table S2. Nucleotide sequences of qPCR primers used in this study

Primer name	Sequence (5' to 3')	NCBI accession numbers
β-actin Forward	CACCACCACAGCCGAAAGAG	EF026002.1
β-actin Reverce	ACCGCAAGATTCCATACCCA	
gpx4a Forward	TGCGACACCACAACTGACTGAAC	NM_001346537.1
gpx4a Reverce	ACCAATGATCCCACTGCTCGATAAAG	
gpx4b Forward	CGCTGAGAAGGGTTTACGCATCC	NM_001030070.2
gpx4b Reverce	CTCCTTAATCTCCGCTTCACTTCCAG	
hamp1 Forward	TGTTTCTGGCTGCTGTCGTCATC	NM_205583.2
hamp1 Reverce	GATGCTCATCCTGTACCTGCTGTATG	
hamp2 Forward	CAGATCACAGCCGTTCCCTTCATAC	NM_001289794.1
hamp2 Reverce	TTCAGCTTCAGTCAGATGTTGGTTCTC	
nrf2 Forward	TCGGGTTTGTCCCTAGATG	NM_182889.1
nrf2 Reverce	AGGTTTGGAGTGTCCGCTA	
keap1 Forward	CCAACGGCATAGAGGTAGTTAT	NM_182864.2
keap1 Reverce	CCTGTATGTGGTAGGAGGGTT	
il-6 Forward	TCAACTTCTCCAGCGTGATG	NM_001261449.1
il-6 Reverce	TCTTTCCCTCTTTTCCTCCTG	
il1-b Forward	TGGCGAACGTCATCCAAG	NM_212844.2
il1-b Reverce	GGAGCACTGGGCGACGCATA	
nfκb Forward	GGCAGGTGGCGATAGTGTT	NM_001001839.2
nfκb Reverce	CATTCCTTCAGTTCTCTTGCG	
ptgs2 Forward	TGGATCTTTCCTGGGTGAAGG	NM_153657
ptgs2 Reverce	GAAGCTCAGGGGTAGTGCAG	
ho-1 Forward	ATGCCCTTGTTTCCAGTCAGC	NM_205671.1
ho-1 Reverce	GGACTTGGAGCACTTCTTCGG	
fth1 Forward	CAGGACGTGAAGAAACCAGAGAAGG	NM_131585.1
fth1 Reverce	ATGTGAGGGTCGTTGTGTTGAGATG	
